# Digitally Enabled Peer Support Intervention to Address Loneliness and Mental Health: Prospective Cohort Analysis

**DOI:** 10.2196/48864

**Published:** 2023-11-06

**Authors:** Dena M Bravata, Joseph Kim, Daniel W Russell, Ron Goldman, Elizabeth Pace

**Affiliations:** 1 Center for Primary Care and Outcomes Research Stanford University San Francisco, CA United States; 2 Wisdo Health, Inc New York, NY United States; 3 Department of Human Development & Family Studies Iowa State University Ames, IA United States; 4 Department of Technology Management and Innovation Tandon School of Engineering New York University New York City, NY United States; 5 Peer Assistance Services, Inc Denver, CO United States

**Keywords:** peer-support, social isolation, loneliness, companionship, depression, anxiety, quality of life, occupational health

## Abstract

**Background:**

Social isolation and loneliness affect 61% of US adults and are associated with significant increases in excessive mental and physical morbidity and mortality. Annual health care spending is US $1643 higher for socially isolated individuals than for those not socially isolated.

**Objective:**

We prospectively evaluated the effects of participation with a digitally enabled peer support intervention on loneliness, depression, anxiety, and health-related quality of life among adults with loneliness.

**Methods:**

Adults aged 18 years and older living in Colorado were recruited to participate in a peer support program via social media campaigns. The intervention included peer support, group coaching, the ability to become a peer helper, and referral to other behavioral health resources. Participants were asked to complete surveys at baseline, 30, 60, and 90 days, which included questions from the validated University of California, Los Angeles Loneliness Scale, Patient Health Questionnaire 2-Item Scale, General Anxiety Disorder 7-Item Scale, and a 2-item measure assessing unhealthy days due to physical condition and mental condition. A growth curve modeling procedure using multilevel regression analyses was conducted to test for linear changes in the outcome variables from baseline to the end of the intervention.

**Results:**

In total, 815 ethnically and socially diverse participants completed registration (mean age 38, SD 12.7; range 18-70 years; female: n=310, 38%; White: n=438, 53.7%; Hispanic: n=133, 16.3%; Black: n=51, 6.3%; n=263, 56.1% had a high social vulnerability score). Participants most commonly joined the following peer communities: loneliness (n=220, 27%), building self-esteem (n=187, 23%), coping with depression (n=179, 22%), and anxiety (n=114, 14%). Program engagement was high, with 90% (n=733) engaged with the platform at 60 days and 86% (n=701) at 90 days. There was a statistically (P<.001 for all outcomes) and clinically significant improvement in all clinical outcomes of interest: a 14.6% (mean 6.47) decrease in loneliness at 90 days; a 50.1% (mean 1.89) decline in depression symptoms at 90 days; a 29% (mean 1.42) reduction in anxiety symptoms at 90 days; and a 13% (mean 21.35) improvement in health-related quality of life at 90 days. Based on changes in health-related quality of life, we estimated a reduction in annual medical costs of US $615 per participant. The program was successful in referring participants to behavioral health educational resources, with 27% (n=217) of participants accessing a resource about how to best support those experiencing psychological distress and 15% (n=45) of women accessing a program about the risks of excessive alcohol use.

**Conclusions:**

Our results suggest that a digitally enabled peer support program can be effective in addressing loneliness, depression, anxiety, and health-related quality of life among a diverse population of adults with loneliness. Moreover, it holds promise as a tool for identifying and referring members to relevant behavioral health resources.

## Introduction

In the landmark 2023 Advisory on loneliness, isolation, and social connection, the US Surgeon General warned about the public health crisis that loneliness, isolation, and disconnection pose to the American public [1]. Prior to the COVID-19 pandemic, 61% of all US adults reported feeling lonely [2,3] and nearly 72% of Medicaid beneficiaries reported being lonely [4]. From the years 1976 to 2019, the rate of loneliness has increased yearly [1,5]. During the pandemic, people of all ages, in all countries, experienced unprecedented social isolation [6-8]. Social isolation is the objective lack of interaction with others (as happens when people live alone) [9]. Loneliness is a similar yet distinct concept, referring to the subjective, unwelcome feeling of being alone or the gap between one’s expectations of the quantity and quality of social relationships and what is actually experienced [10]. Loneliness and social isolation are associated with significantly decreased capacity for self-care [11,12]; lower quality of life [13,14]; and increases in physical and mental health morbidity and mortality including depression, anxiety, cognitive impairment, coronary heart disease, and stroke [15-27]. Moreover, loneliness is associated with increased health care use including a 15% increase in outpatient physician and emergency room visits and a 36% increase in inpatient admissions compared to nonlonely populations [13,28].

The impact of social isolation and loneliness on health care spending is enormous, with Medicare spending US $6.7 billion annually due to social isolation [14]. On average, health care spending is US $1643 higher annually for socially isolated individuals compared to those who are not socially isolated [29]. Employers bear a significant burden of excessive costs associated with social isolation and loneliness, given that loneliness typically increases in midlife and affects working adults [30-32]. Employees who report feelings of loneliness are twice as likely to miss work due to illness, be less productive, and quit their jobs as those who are not lonely [13]. Lonely workers report, on average, 5.7 more days of absenteeism per year compared to nonlonely employees [3]. The total annual costs of avoidable absenteeism have been calculated as US $154 billion, with an average annual cost per lonely employee of US $1590 [3].

Several interventions have been evaluated for their effectiveness in reducing loneliness and its associated psychological distress [33]. Effective strategies typically include 4 features such as providing emotional support (resulting in the feeling “I am seen and heard”), supporting a sense of worth (resulting in the feeling “I am valued”), supporting a sense of belonging (resulting in the feeling “I belong”), and developing reliable alliance (resulting in the feeling “I can rely on others and that others can rely on me”) [34]. A recent meta-analysis found that a reminiscence intervention was effective; however, there was only 1 study supporting this finding [35]. Disappointingly, cognitive behavioral therapies, which are available to many commercially insured and other covered populations, were found to have the smallest effect size in alleviating loneliness [35]. Some evidence points to peer support as an effective, scalable, and economical solution for addressing various mental health issues but it has not been widely studied for loneliness [36-39].

Peer support is defined as the interpersonal connection based on shared life experiences characterized by empathy and validation [36]. Peer support has been associated with increased engagement in self-care; improved quality of life; and reduced substance use, depression symptoms, and hospital admission rates for some mental health disorders [40,41]. In addition, a meta-analysis examining the effectiveness of digital peer support found that the use of technology to facilitate the delivery of peer support is feasible and effective [38,42]. Thus, the objective of this study was to evaluate the effects of participation using a digital peer support intervention on loneliness, depression, anxiety, and quality of life among adults who are lonely.

## Methods

### Recruitment

Adult participants aged 18 years and older living in Colorado were recruited via social media campaigns on Facebook and TikTok between January and April 2022 to participate in a peer support program developed by Wisdo Health. The ads included text, images, and videos describing the Wisdo peer support app [43] as a safe digital community where participants who feel alone can connect with others going through similar life experiences. Participants were offered 1 year of free access to the digital peer support platform but were not otherwise compensated for their participation.

### Measures

#### Overview

Participants were asked to complete 4 surveys such as when joining the program (baseline) and then after 30, 60, and 90 days of peer support. All surveys were administered within the app. Each survey was available for participants to complete during a 10-day window. We collected demographics at baseline and used the member’s zip codes to calculate their social vulnerability index according to the Centers for Disease Control and Prevention [44], which accounts for 16 social factors including poverty, lack of vehicle access, and crowded housing. The following measures were included in the surveys.

#### University of California, Los Angeles (UCLA-3) Loneliness Scale

This validated measure of loneliness [45] includes the following questions: How often do you feel that you lack companionship? How often do you feel left out? How often do you feel isolated from others? For each question, participants were asked to choose among 1=hardly ever, 2=some of the time, and 3=often. A total score of 4 or greater is considered positive for loneliness, and a score of 7 to 9 is considered severely lonely.

#### Patient Health Questionnaire 2-Item Scale

The Patient Health Questionnaire 2-Item Scale (PHQ-2) is a validated measure [46] that asks about the frequency of depressed mood and anhedonia over the past 2 weeks. Items are scored from 0 (not at all) to 3 (nearly every day). A total score of 3 or greater indicates that a major depressive disorder is likely.

#### General Anxiety Disorder 7-Item Scale

A single item from the validated General Anxiety Disorder 7-Item Scale (GAD-7) [47] was used: “Over the past 2 weeks, how often have you been bothered with feeling nervous, anxious, or on edge?” This item was scored from 0 (not at all) to 3 (nearly every day).

#### Unhealthy Days

This validated measure captures information on the physical and mental health status of individuals and on the impact of health status on quality of life [48]. It includes 2 questions: “Thinking about your physical health, which includes physical illness and injury, for how many days during the past 30 days was your physical health not good?” and “Thinking about your mental health, which includes stress, depression, and problems with emotions, for how many days during the past 30 days was your mental health not good?” The 2-item measure assessing physically and mentally unhealthy days is highly correlated with traditional measures of health, including morbidity, mortality, and health care costs. Each additional unhealthy day is associated with an incremental cost increase of US $15.64 per member per month [49].

#### Program Satisfaction

Participants were asked whether they would recommend the program to others, whether they believe that the program should be made available to everyone in Colorado, and to provide subjective comments.

### Intervention

#### Overview

**Figure 1:**
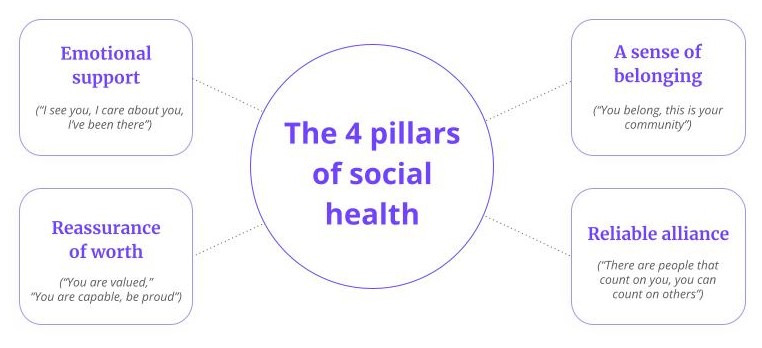
The 4 pillars of social health: The 4 pillars of social health are derived from the Social Provisions Scale [53].

The Wisdo intervention is a peer support and social health platform designed to enable the 4 pillars of social health such as emotional support, reassurance of worth, a sense of belonging, and reliable alliance (Figure 1) [34,50-53]. The platform is available as a native app on iOS and Android devices and as a web app on personal computers and is HIPAA (Health Insurance Portability and Accountability Act) and System and Organization Controls (SOC) 2 + HITRUST (Health Information Trust) compliant. Members can be anonymous on the platform as they are not asked to use their real names. Since its launch in 2018, over 500,000 adults aged 17 to 80 years have participated in the platform’s peer support communities. There are 5 key aspects to the peer support intervention such as self-mapping, connecting with peers, group coaching, referrals to other behavioral health resources, and the ability to become a peer helper.

#### Self-Mapping

**Figure 2:**
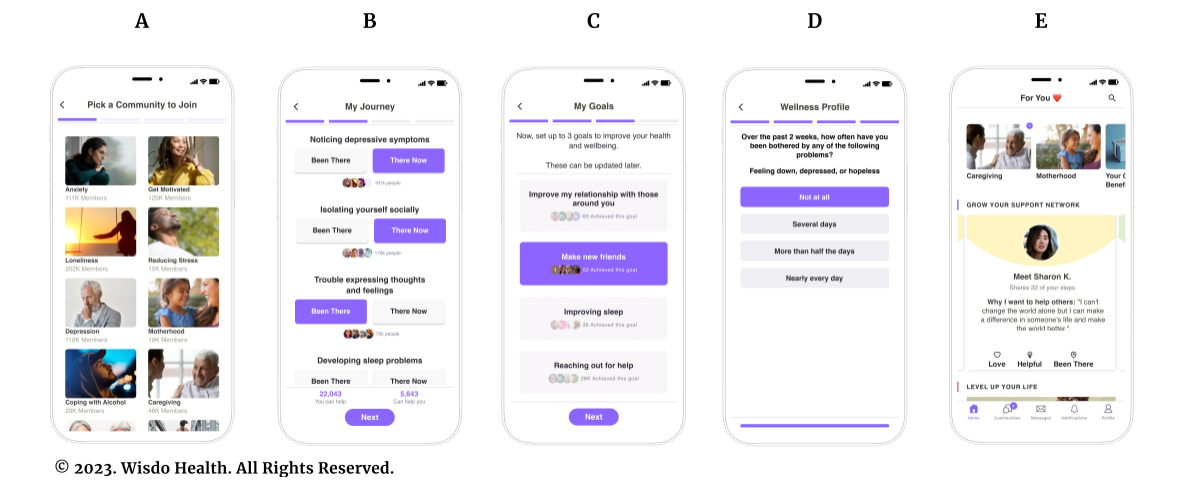
Program application: new members are on-boarded to the platform with a self-mapping exercise, which includes 4 steps. The first step (A) is to select the first community they want to join based on a topic they are concerned about and that makes them feel most lonely. In the second step (B), members are asked to identify as either “been there” or “there now” on a series of 25 to 35 key challenges, obstacles to health, gaps in care, and milestones that are frequently experienced by individuals in their selected community. In the third step (C), members choose up to 3 personal goals from a list of 10. In the fourth step, members answer brief, validated measures (D). The members are connected to specific peers based on shared lived experience and how helpful those peers have been to similar members (E).

New members are onboarded to the platform with a self-mapping exercise that is critical for assigning the participant to a curated social support community comprised of helpful peers with shared lived experiences. The self-mapping element includes 4 steps (Figures 2A-2E). First, members select the community they want to join based on a topic they are concerned about and that makes them feel most lonely. Members choose from over 50 communities covering areas such as behavioral health (eg, anxiety, depression, loneliness, and alcohol use), physical health (eg, diabetes, heart failure, and cancer), family (eg, caregiver and parenthood), workplace (eg, burnout, stress, and working remotely), and self-growth (eg, positive thinking and starting to exercise; Figure 2A). On average, members join 6 communities during their first year on Wisdo.

Second, members are asked to identify as either “been there” or “there now” on a series of 25 to 35 key challenges, obstacles to health, gaps in care, and milestones that are frequently experienced by individuals in their selected community (Figure 2B). On average, members click on 20 such milestones per community they join, providing in-depth insights into their experiences to date. As members click through each step, they can see how many people on the platform have clicked the same step, further promoting 2 important pillars of social health such as a sense of belonging and emotional support.

Third, members choose up to 3 personal goals from a list of 10 (eg, making new friends, better managing stressful events, and improving sleep) relevant to their community (Figure 2C). As members click on a goal, they can see how many other members on the platform have already accomplished it. Members can track their progress toward achieving each goal.

Finally, members answer brief validated measures (Figure 2D). These responses establish a baseline for members’ social and mental health and enable them to track changes over time.

As members continue to join additional communities, establish goals, and answer follow-up surveys, the self-mapping information shared by members during onboarding is used by the platform’s artificial intelligence engine to create curated connections with helpful peers, identify members to join group coaching sessions to develop social health skills, and connect members with covered clinical programs and services to support social barriers to care.

#### Connecting With Peers

The peer support platform continuously suggests connections with helpful peers based on the number of shared steps (ie, “been there” and “there now”) and how helpful these peers have been to similar participants. Once connected, participants can chat on the app one-on-one and in group settings and receive emotional, nonjudgmental support and encouragement (Figure 2E). They can also share “empathy-charged” reactions to posts by other members that build up emotional support and a sense of worth, such as “Helpful,” “Love,” and “Been There.” The community is moderated by trained staff augmented by its artificial intelligence engine, which monitors over 600 keywords and phrases that could indicate a potential risk. Members who fail to follow the program’s code of conduct are warned and may be banned from the platform.

#### Group Coaching

Members can join weekly group video coaching sessions conducted by certified life coaches via video. For anonymity, participants can choose not to turn on their video cameras. Each group coaching topic allows members to connect around a shared goal and skill (eg, building emotional resilience, developing a sense of purpose, and developing a sense of self-worth) or interest (eg, travel, books, and exercise). Together, this support from peers and groups provides the key elements of a loneliness intervention, namely emotional support, reassurance of worth, a sense of belonging, and reliable alliance.

#### Referral to Other Behavioral Health and Social Determinants of Health Benefits Programs

The program matches participants to covered benefits and community services offered by their employer, health plan, government agencies, or local nonprofits. Once a participant is flagged as a candidate for a covered benefit, the program deploys a combination of several tactics, including in-app messages, push notifications, and an invitation to relevant coaching sessions to inform and motivate the member to engage with the relevant covered benefit.

#### Ability to Become a Peer Helper

Highly rated members who are frequently considered helpful by other community members are invited to enroll in a web-based training program to earn a Helper badge. The program includes 5 modules covering topics such as providing emotional support, motivational interviewing skills, and caring for oneself. Each module is followed by a quiz. Users who successfully complete the training are awarded the Helper badge and provided with an explanation of how their performance will be continuously monitored and assessed to ensure safety and quality on the platform. On average, 10%-20% of members joining the peer support platform successfully complete the training and earn the Helper badge. This format promotes 2 important pillars of social health such as reliable alliance and reassurance of worth.

### Statistical Analysis

We computed means at baseline for each of the outcomes of interest. Since not all participants completed follow-up surveys at each of the measurement periods, we compared the baseline scores on the outcome measures for individuals who did and did not complete the subsequent surveys. The results indicated that none of the differences in the outcome measures from the baseline survey differed significantly between individuals who did or did not complete the subsequent surveys.

To conduct the analyses of change and include all cases in the analyses, we tested for change over time on the outcome variables using a growth curve modeling procedure via multilevel regression analyses. We tested for linear changes in the outcome variables and included a predictor variable that reflected whether the individual had complete data over time across the 3 surveys. The inclusion of this last predictor variable allowed us to test whether the pattern of change on the outcome variable varied for participants with complete data across the 3 surveys versus participants with only partial data.

### Ethical Considerations

Given that all data used in this analysis were routinely collected as part of standard program participation and were deidentified, this protocol was considered exempt from human subjects’ consent (WCG IRB protocol Wisdo.001; January 26, 2023).

## Results

### Participant Characteristics

Overall, 4500 people clicked on a recruitment ad, 1141 installed the app on their smartphones, and 815 completed the registration process and joined the study. The average age of participants was 38 (SD 12.7; range: 18-70) years, 310 (38%) identified as female, 438 (53.7%) identified as White, 133 (16.3%) as identified Hispanic, 51 (6.3%) as identified Black, and 263 (56.1%) were categorized as having a high social vulnerability score (Table 1).

**Table 1:** Demographic characteristics of sample participants.^a^

Characteristics		Values
**Age (years) (N=815), n (%)**		
	18-25	122 (15)
	26-34	212 (26)
	35-44	236 (29)
	45-54	139 (17)
	55-64	82 (10.1)
	65 and older	24 (2.9)
**Gender (N=815), n (%)**		
	Female	310 (38)
	Male	473 (58)
	Nonbinary	16 (2)
	Other	16 (2)
**Race (N=815), n (%)**		
	Asian	19 (2.3)
	American Indian	21 (2.6)
	Black	51 (6.3)
	Hispanic	133 (16.3)
	White	438 (53.7)
	Other	153 (18.8)
**Social Vulnerability Index (N=469), n (%)**		
	High	263 (56.1)
	Medium	2 (0.4)
	Medium-low	41 (8.7)
	Low	163 (34.9)

^a^The table displays the demographic characteristics of the sample participants. The participants represent diverse groups with the majority having a high score on the social vulnerability index.

### Engagement With Peer Support

On average, participants joined 6 communities during the pilot period. The most commonly selected communities were loneliness (n=220, 27%), building self-esteem (n=187, 23%), coping with depression (n=179, 22%), anxiety (n=114, 14%), exercising regularly (n=65, 8%), and coping with alcohol addiction (n=49, 6%; Table 2).

When joining a community, on average, participants picked 16.3 (SD 4.3) “there now” indications and 12.0 (SD 3.8) “been there” during their self-mapping process. Overall, 71% (n=579) of participants reported “there now” steps associated with behavioral health challenges, 32% (n=261) with sleep problems, and 13% (n=106) with nutrition challenges. On average, members chose 2.3 (SD 0.46) goals (the maximum allowed was 3). The most common goals were making new friends (n=228, 28%), improving my relationships with those around me (n=147, 18%), and avoiding negativity (n=130, 16%; Table 3).

On average, participants visited the platform 3 times a month, sent 40 messages, and established 10 new meaningful connections, defined as connections where both users interacted in a back-and-forth conversation on the platform. Throughout the study, engagement in the platform remained high, with 90% (n=733) engaged with the platform after 60 days and 86% (n=701) engaged with the platform after 90 days. Many members viewed messages from peers (n=701, 86%) and groups (n=652, 80%). Members were also active in sending private or group messages (n=399, 49%). Overall, 68% (n=554) of registered users engaged in conversations with a peer within their first month on the platform, 43.9% (n=358) during their second month, and 32% (n=261) during their third month. Members who received the Helper badge reported a high percentage of engagement in conversation, with 50% (n=61) of them engaged in conversation during their third month.

**Table 2:** Commonly selected communities (N=815).^a^

Community name	Participants, n (%)
Loneliness	220 (27)
Building self-esteem	187 (23)
Coping with depression	179 (22)
Anxiety	114 (14)
Exercising regularly	65 (8)
Coping with alcohol addiction	49 (6)
Coping with substance use	24 (3)
LGBTQIA^b^	24 (3)
Coping with loss	16 (2)
Coping with drug addiction	8 (1)

^a^The most commonly selected communities were loneliness and building self-esteem.

^b^LGBTQIA: lesbian, gay, bisexual, transgender, queer, intersex, and asexual.

**Table 3:** Commonly reported goals of participants (N=815).^a^

Goal	Participants, n (%)
Making new friends	228 (28)
Improve my relationships with those around me	147 (18)
Avoiding negativity	130 (16)
Being more accepting	98 (12)
Improving my sleep	82 (10)
Being able to better manage stressful events	73 (9)
Avoiding comparisons with others	65 (8)
Have a positive impact on the world	65 (8)
Learning mindfulness	65 (8)
Start to exercise and stick with it	65 (8)
Improve my diet	65 (8)
Finding like-minded support	49 (6)
Reduce alcohol or other drug consumption	49 (6)
Helping others	41 (5)
Practicing forgiveness	41 (5)

^a^The most commonly cited goals for peer support were making new friends and improving relations with others.

### Referrals to Behavioral Health Resources

In total, 27% (n=217) percent of participants clicked the link to access an educational module about how to support peers experiencing alcohol misuse or psychological distress. Additionally, 15% (n=45) of all female members clicked the link to access an educational module on the risks of excessive alcohol consumption for women.

### Clinical Outcomes

#### Overview

Of the 815 participants, 595 completed the baseline survey, and 130 completed one or more of the subsequent surveys.

#### Loneliness

The average loneliness score at baseline was 7.41 (SD 1.65), with 98% (n=583) screening positive for loneliness (UCLA-3 Loneliness Scale score of 4-9) and 68.7% (n=409) screening as severely lonely (UCLA-3 Loneliness Scale score of 7-9). After 90 days, 41.5% (n=54) of individuals who screened as severely lonely (UCLA-3 Loneliness Scale score of 7-9) when joining the study had lower loneliness scores and 4.6% (n=6) scored as no longer lonely (UCLA-3 Loneliness Scale score of 3). Overall, 10.4% (n=13) individuals who screened as lonely when joining the study (UCLA-3 Loneliness Scale score of 4-9) scored as not lonely after 90 days (UCLA-3 Loneliness Scale score of 3) and 31.5% (n=41) reported no change in their loneliness level.

**Figure 3:**
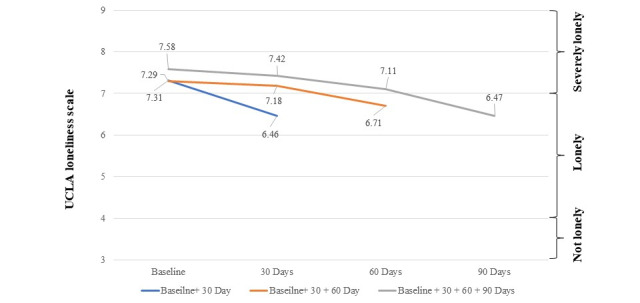
Change in Loneliness: UCLA Loneliness scores between 3 and 4 indicate nonlonely participants. Scores between 4 and 7 indicate lonely individuals. Scores between 7 and 9 indicate severely lonely individuals. The average score for participants with complete data (gray line) and incomplete data (orange and blue lines) indicates that the groups were all severely lonely. The participants who only reported loneliness at baseline and at 30 days had the greatest improvement in loneliness (blue line). The change in loneliness over time did not differ for participants with complete data (gray line) versus incomplete data (orange and blue lines). UCLA-3: University of California, Los Angeles.

There was a statistically significant decrease over time in levels of loneliness (*b*=–0.01; *t*_197_=–5.65; P<.001; Figure 3). Overall, loneliness decreased 11.6% (mean 6.46) within the first 30 days and 14.6% (mean 6.47) between the baseline and 90-day assessments. The participants who only reported loneliness at baseline and at 30 days had the greatest improvement in loneliness. The change over time in loneliness did not differ significantly between participants with complete data versus individuals with only partial survey data (*t*_196_=0.62).

#### Depression

**Figure 4:**
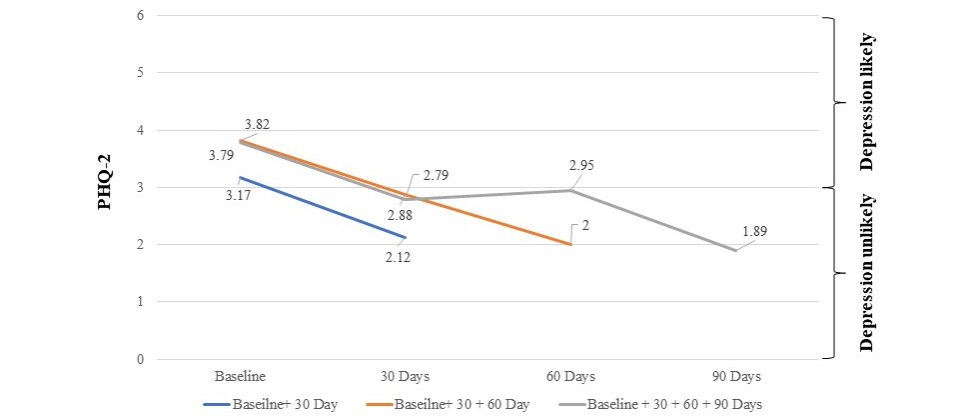
Change in depression: PHQ-2 scores between 0 and 3 indicate a major depressive disorder is unlikely and PHQ-2 scores between 3 and 6 indicate a major depressive disorder is likely. The average scores at baseline for participants with complete data (gray line) and incomplete data (orange and blue lines) indicate that all groups were likely to have a major depressive disorder. Upon completion of 1 assessment after 30 days, all groups observed a decline in PHQ-2 scores and were unlikely to have depression. PHQ-2: Patient Health Questionnaire 2-Item Scale.

The average depression score at baseline was 3.49 (SD 1.91). In total, 61% (n=363) of participants joined the study with a score of 3 or higher (ie, screened at risk for depression); of these, 65% (n=236) reported below-risk levels of depression (<3 on PHQ-2) within 30 to 90 days. Overall, there was a 33.1% (mean 2.12) decline in depression symptoms by 30 days and a 50.1% (mean 1.89) decline in depression symptoms between baseline and day 90 (Figure 4; *b*=–0.02; *t*_197_=–6.48; P<.001). This decline did not vary as a function of whether the participant completed all 4 surveys (*t*_196_=–0.40).

#### Anxiety

**Figure 5:**
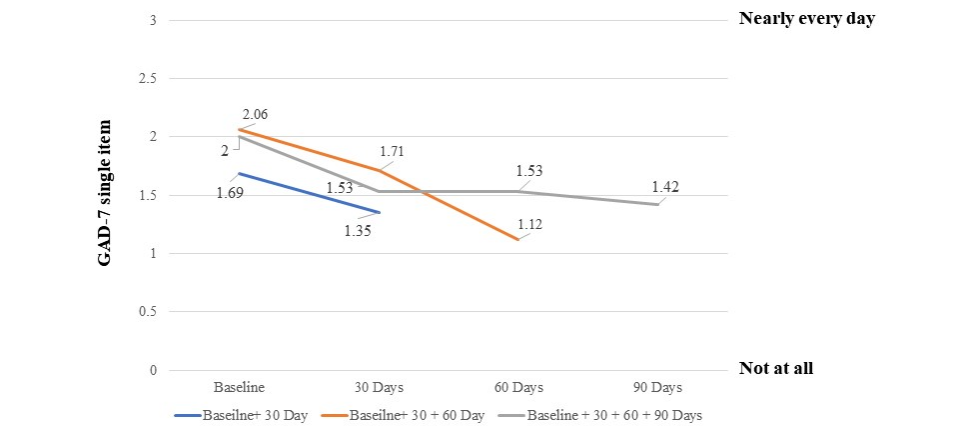
Change in anxiety: the average GAD-7 single-item score is displayed on the y-axis and the assessment of anxiety at 30-day intervals is on the x-axis. The average anxiety score declined over time for individuals with complete data (gray line) and incomplete data (orange and blue lines); however, they did not differ significantly. GAD-7: General Anxiety Disorder 7-Item Scale.

The average anxiety score at baseline was 1.86 (SD 1.00), with 87.9% (n=523) of participants reporting feeling nervous, anxious, or on the edge for at least several days over the past 2 weeks and 30% (n=179) saying they experience these feelings nearly every day. Participants had a 20.1% (mean 1.35) reduction in anxiety symptoms by day 30 and a 29% (mean 1.42) reduction in anxiety from the baseline to day 90 (Figure 5). There was a statistically significant decline in levels of anxiety over time (*b*=–0.16; *t*_197_=–3.42; P<.001). This pattern of results did not appear to vary for participants who provided complete data versus individuals who did not complete all the surveys (*t*_196_=–0.18).

#### Health-Related Quality of Life

The average number of unhealthy mental health days at baseline was 20.1 (SD 9.9) days, and the average number of unhealthy physical health days at baseline was 15.9 (SD 11.4) days. At baseline, 88.9% (n=529) reported having 6 or more mentally unhealthy days in the prior month and 52.9% (n=315) reported having 6 or more physically unhealthy days during the same period. There was a statistically significant (13%) improvement in health-related quality of life from the baseline survey (mean 24.63, SD 10.34) to the 90-day survey (mean 21.35, SD 9.13; *t*_39_=2.02; P=.05). On average, participants reported 3.28 less monthly unhealthy days after 90 days on the platform. This indicates the potential of the platform to drive an annual reduction of medical costs equaling US $615 per participant (3.28 days×US $15.64 estimated cost reduction per unhealthy day×12 months).

#### Program Satisfaction

Overall, 88% (n=91) reported that they would recommend the peer support platform to others, and 98% (n=102) of participants said that they believed that the program should be made available to everyone in Colorado. In subjective comments, participants frequently wrote that being able to connect and talk with others who have shared lived experiences in a safe, judgment-free, and supportive environment was the most valuable part of their experience. No participants wrote a negative comment.

## Discussion

This study of the use of a novel peer-support platform by a demographically diverse population demonstrated 5 key findings. First, adults of a wide range of ages, genders, and social vulnerability seek and remain engaged with digitally enabled peer support for loneliness. Throughout the study, engagement in the platform remained high, with 86% (n=701) engaged in the platform after 90 days. This is significantly higher compared to digital community–driven applications, which on average have a 19% retention rate after 90 days, and mental health apps which, on average, have a 3% retention rate after 30 days [54,55]. Relatedly, participants seemed highly satisfied with the digital peer support program, with 88% (n=91) of participants recommending that it be made available to others. Men have been observed to be less likely to seek support for mental health compared to women [56]. The high rate of participation (n=473, 58%) and engagement in males point to the effectiveness of the program in drawing men seeking peer support. With minorities and underrepresented populations reporting higher than average rates of loneliness, the results of this study also suggest that the platform can increase the outcomes of diversity equity and inclusion initiatives implemented by government agencies and employers [3,43].

Second, participation in peer support was associated with a significant reduction in loneliness with an 11.6% (mean 6.46) improvement within the first 30 days, increasing to 14.6% (mean 6.47) by day 90. Loneliness occurs at all stages of the life span with young adults reporting the highest mean levels of loneliness and older adults also reporting high levels of loneliness [57,58]. Interventions to address loneliness and social isolation have historically been observed to reduce loneliness within a short time period, however, with a small effect size [59]. The results of this study suggest that a digitally enabled peer support platform can be an effective approach to mitigating loneliness, which has become a prominent issue due to its high prevalence in the United States [3,4,14].

Third, given that loneliness is often comorbid with depression and anxiety, a key finding of this study was that participants reported significant improvements in depression and anxiety symptoms (50.1%, mean 1.89 and 29%, mean 1.42, respectively, at 90 days). This promising finding suggests that as employers and health plans seek interventions to increase access to behavioral health services for their populations, peer support should be considered among the solution set. Moreover, these findings should be evaluated in future studies with full Patient Health Questionnaire-9 and GAD-7 instruments.

Fourth, participants reported a 13% (mean 21.35, SD 9.13) reduction in the number of monthly mentally and physically unhealthy days after 90 days on the platform when compared to baseline. The results of the platform point to the importance of peer support in improving clinical outcomes and improving the quality of life of participants. The magnitude of this improvement may have partially been a result of the use of the 2-item measure assessing physically and mentally unhealthy days compared to the original, long form of the health-related quality of life. Due to the large number of questions already included in the participant survey, the short 2-item form was used in this study. A future study will include the original health-related quality of life instrument or similar instruments (eg, EQ-5D or the 36-Item Short Form Survey [SF-36]).

At US $15.64 per unhealthy day, one could estimate that participation in peer support was associated with a US $615 reduction in annual medical costs. This finding warrants further exploration with a detailed economic analysis of participation with digitally enabled peer support for lonely populations.

Finally, the platform was successful in referring participants to mental health education resources, with 27% (n=217) of participants accessing a resource about how to best support those experiencing psychological distress and 15% (n=45) of women accessing a program about the risks of excessive alcohol use. This finding suggests that a peer support platform can be applied to effectively identify, motivate, and connect appropriate users with community services, clinical programs, and health literacy resources. Given the challenges of engaging members with valuable employer- and health plan–sponsored programs for which they are eligible, the role of a digitally enabled peer-support platform as a benefit navigation and engagement tool warrants future study.

The implication of this study is that a digitally enabled peer support platform is a resource for addressing the epidemic of loneliness, depression, and anxiety. A significant consideration for clinicians is systematic screening to identify adults who are lonely and to refer them for enrollment in the platform. Health insurance providers could consider covering fees associated with participation in the platform for patients who screen positive for loneliness using validated instruments. The costs associated with loneliness are significant. Thus, providing access to a scalable digitally enabled peer support platform could be a cost-effective or perhaps even cost-saving intervention.

This study had 3 key limitations. First, all participants were adults from Colorado. While there is no reason to suspect that clinical characteristics of adults with loneliness in Colorado vary significantly from those elsewhere in the United States, future studies should evaluate geographically diverse populations. Second, this study did not have a control group, and our statistical power was limited by the number of participants who completed the surveys at all time points. A randomized controlled trial that compares peer support to other interventions available to similar populations (such as cognitive behavioral therapy) would be a valuable contribution to the literature. Finally, we did not separately evaluate each of the pillars of the intervention: “emotional support, reassurance of worth, a sense of belonging, and reliable alliance.” Each of these pillars is a component of the Social Provisions Scale [53], and future studies analyzing the factors of social provisions will be an important area to examine.

The Surgeon General recently wrote [1]:

Loneliness and isolation represent profound threats to our health and well-being. But we have the power to respond. By taking small steps every day to strengthen our relationships, and by supporting community efforts to rebuild social connection, we can rise to meet this moment together.

We believe that digitally enabled peer support represents a valuable evidence-based tool for creating meaningful social connections for a wide range of at-risk populations.

## Abbreviations

GAD-7: General Anxiety Disorder 7-Item Scale
HIPAA: Health Insurance Portability and Accountability Act
HITRUST: Health Information Trust
PHQ-2: Patient Health Questionnaire 2-Item Scale
SF-36: 36-Item Short Form Survey
UCLA-3: University of California, Los Angeles Loneliness Scale

## Data Availability

Data used in this analysis will be made available upon reasonable request to the corresponding author.
